# Protection of cells from salinity stress by extracellular polymeric substances in diatom biofilms

**DOI:** 10.1080/08927014.2014.960859

**Published:** 2014-09-30

**Authors:** Deborah J. Steele, Daniel J. Franklin, Graham J.C. Underwood

**Affiliations:** ^a^ School of Biological Sciences, University of Essex, Colchester, UK; ^b^ Faculty of Science & Technology, Bournemouth University, Poole, UK; ^c^ Plymouth Marine Laboratory, Plymouth, UK

**Keywords:** biofilm, diatoms, *Cylindrotheca*, exopolymer, sea ice, salinity, xanthan gum

## Abstract

Diatom biofilms are abundant in the marine environment. It is assumed (but untested) that extracellular polymeric substances (EPS), produced by diatoms, enable cells to cope with fluctuating salinity. To determine the protective role of EPS, *Cylindrotheca closterium* was grown in xanthan gum at salinities of 35, 50, 70 and 90 ppt. A xanthan matrix significantly increased cell viability (determined by SYTOX-Green), growth rate and population density by up to 300, 2,300 and 200%, respectively. Diatoms grown in 0.75% w/v xanthan, subjected to acute salinity shock treatments (at salinities 17.5, 50, 70 and 90 ppt) maintained photosynthetic capacity, *F*_*q*_ *′/F*_*m*_ *′*, within 4% of pre-shock values, whereas *F*_*q*_ *′/F*_*m*_ *′* in cells grown without xanthan declined by up to 64% with hypersaline shock. Biofilms that developed in xanthan at standard salinity helped cells to maintain function during salinity shock. These results provide evidence of the benefits of living in an EPS matrix for biofilm diatoms.

## Introduction

Microbial photosynthetic biofilms consist of prokaryotic and eukaryotic phototrophs embedded in a matrix of extracellular polymeric substances (EPS). Biofilms occur widely in almost all terrestrial and aquatic environments and provide important ecosystem functions, including sediment stabilisation (Paterson 1997; Lan et al. 2014), primary production (Underwood & Kromkamp 1999) and nutrient cycling (Nedwell et al. 1999), but also cause fouling on artificial maritime surfaces (eg Molino & Wetherbee 2008; Mieszkin et al. 2013). In many environments, such as intertidal sediment flats (Rothrock & Garcia-Pichel 2005; McKew et al. 2011), sea ice (Gleitz & Thomas 1993; Krembs et al. 2011), ships’ hulls, soil and desert biocrusts (Lan et al. 2014), salt marshes (Underwood 1997) and glacial surfaces (Yallop et al. 2012), biofilms are exposed to fluctuating (and in some cases, extreme) conditions of water availability and salinity (Gleitz & Thomas 1993; Underwood 1997; McKew et al. 2011). It has long been speculated that the ubiquity of EPS in biofilms is partly due to microorganisms modifying their immediate surroundings to reduce the negative impacts of water stress by changing the production and composition of their EPS (Decho 1990; Krembs & Deming 2008). This microscale environmental buffering by microbes has macroscale consequences (Decho 1994) as it allows the physical development and succession of primary producer assemblages in harsh environments, for example in large scale stabilisation of desert soils by cyanobacterial filaments and EPS (Lan et al. 2014), biostabilisation of marine sediment habitats (Yallop et al. 2000), and in the structure and organic carbon composition of polar sea ice (Krembs et al. 2011; Underwood et al. 2013).

Low or fluctuating water potentials are known to stimulate the production of EPS by bacteria (Dudman 1977; Roberson & Firestone 1992); the EPS provides a beneficial, hydrated microenvironment around the cell (Tonn & Gander 1979; Roberson & Firestone 1992; Chang & Halverson 2003). However, this process has received much less attention in microalgae. As microalgae, and in particular diatoms, produce EPS, an equivalence between the function of bacterial and microalgal EPS has long been suspected (Hostetter & Hoshaw 1970; Davis 1972).

Benthic diatoms associated with sea ice and intertidal sediment are well adapted to fluctuating conditions and can tolerate a wide range of salinities (Gleitz & Thomas 1993; Clavero et al. 2000; Krell et al. 2008). Some of these diatom species, eg *Cylindrotheca closterium* (Zargiel et al. 2011), are also known to adhere to artificial surfaces and cause biofouling[Bibr CIT0073]. Diatoms can reduce the effects of salt stress by the active transport of ions out of the cell or into the vacuole (Shi et al. 2002) and by the regulation of cellular osmolytes (Clavero et al. 2000). However, these mechanisms are energetically expensive and can therefore halt or slow cell division (Krell et al. 2007). Hence, an EPS-based mechanism that buffers the effects of fluctuating water potential may provide a competitive advantage to diatoms, as it does for bacteria. For example, the benthic diatom *Phaeodactylum tricornutum* increases production of EPS and produces a higher proportion of uronic acids and sulphates at elevated salinities (Abdullahi et al. 2006), potentially allowing the EPS to retain more water. *Nitzschia frustulum* produces more rhamnose and xylose sugars under elevated salinity, causing the EPS gel to become thicker (Allan et al. 1972), which restricts the diffusion of anions towards the cell. The sea-ice diatom *Fragilariopsis cylindrus* increases EPS production at reduced temperatures and elevated salinity. The EPS inhibits ice formation and therefore acts as a cryoprotectant (Aslam et al. 2012). Although these adaptations support the concept that living within an EPS matrix is beneficial, there have been few, if any, direct tests of this hypothesis.

To further understand EPS protection of diatoms, a model system was used that allowed broad conclusions to be reached that are applicable to natural biofilms, which vary substantially in terms of species composition and microstructure. *Cylindrotheca* is a common maritime fouling genus and the species *C. closterium* has been reported in previous biofouling studies (Zargiel et al. 2011; Zargiel & Swain 2014). *C. closterium* is commonly used as a model species for studies of EPS (Staats et al. 1999; de Brouwer & Stal 2002) and previously caused a mucilage event in the Adriatic Sea (Najdek et al. 2005). It is common in mud flats and is known to modify its EPS production with changes in the environment (Alcoverro et al. 2000; Apoya-Horton et al. 2006).

The monosaccharide composition of diatom EPS varies between species but generally glucose, mannose, galactose and rhamnose are the most abundant sugars (Hoagland et al. 1993). *C. closterium* EPS consists of a highly hydrated matrix of strands with glucose and mannose as the dominant monosaccharides, which are acidified to uronic acids (Hoagland et al. 1993; Apoya-Horton et al. 2006), a composition also found in *Navicula* species (Bhosle et al. 1995; Staats et al. 1999). Specifically, *C. closterium* EPS fractions produced under a 12:12 h dark:light cycle contained 82.5% glucose and 7.6% mannose in the non-attached state (removed by centrifugation) (Staats et al. 1999) and in the attached state (extracted in 30°C water for 1 h), 22.9% glucose and 14.7% rhamnose. The prevalence of uronic acids was a common feature measured in the biofouling model species *Craspedostauros australis* and *Amphora coffeaeformis* (Poulsen et al. 2014). This common composition of diatom EPS is similar to xanthan gum, a polysaccharide gel synthesised by the bacterium *Xanthomonas campestris* and widely used as a reference standard for EPS in the marine environment (Passow & Alldredge 1995; Krembs et al. 2011) and for soil biofilms (Hart et al. 2001). Xanthan gum consists of glucose, mannose and glucuronic acid in the ratio 2:2:1 with terminal ends of pyruvate, which is thought to be important in cross-linking the xanthan molecules and so contributing structure to the gel. Pyruvate has also been detected, accounting for 20% of the biofilm EPS of the diatom *Amphora rostrata* (Khandeparker & Bhosle 2001). However, the high proportion of uronic acids in diatom EPS suggests that their molecular cross-linking is due to ionic interactions between divalent cations and the carboxylic group of the uronic acids, as occurs in bacterial biofilms (Sutherland 2001). The similarities between xanthan gum and *C. closterium* EPS make xanthan a useful tool for studying the protective qualities of EPS to cells.

This study investigated how the presence of an EPS matrix can influence the survival of *C. closterium* during growth at standard (35 ppt) and elevated salinities and during salinity fluctuations. For the first time experiments were explicitly designed that directly measured the effects of EPS on diatom cell viability and photosynthetic capacity, both of which govern population growth and persistence. The study examined whether increased EPS concentrations enhanced population growth and long term viability, and whether this effect was greater at higher salinity. The study also examined whether the presence of an EPS matrix protected cells from acute osmotic shock over short time periods.

## Materials and methods

### Culture conditions

*Cylindrotheca closterium* (Reimann et Lewin) from the University of Essex culture collection was grown in an artificial seawater base (35 ppt Reefsalt, AquaWorld, Swallow Aquatics, Southend-on-Sea, UK) enriched with f/2 medium (Guillard 1975). Cultures were maintained at salinity 35 ppt prior to the study and were not acclimated to the experimental conditions. Batch cultures were grown in a Qualicool 260 incubator (LTE Scientific Ltd, Oldham, UK) at 20°C and 24 h light at an irradiance of 12 ± 0.5 μmol photons m^−2^ s^−1^ provided by fluorescent lamps. Stock cultures were treated with gentamicin and penicillin–streptomycin solution (5 μg ml^−l^, Sigma-Aldrich, Dorset, UK), before experimental use, in order to minimise bacterial growth. Aseptic techniques were used throughout all procedures.

### Long-term growth at elevated salinities

Cultures were grown in a 4 × 4 matrix of treatments: salinities of 35, 50, 70 and 90 (ppt/unitless) and xanthan gum (Sigma-Aldrich) at concentrations of 0, 0.04, 0.38 and 0.75% w/v. Salinity was increased from medium salinity (35 ppt) by adding NaCl. Xanthan gum was dissolved overnight into the growth medium and stock culture cells were added at starting population densities of 1.5 × 10^5^ cells ml^−1^; determined by a haemacytometer count (Neubauer, improved bright-line). For treatments at salinities 35, 50 and 70 ppt, 12 sterile 5 ml Petri dishes (Sigma-Aldrich) were inoculated with 4 ml of the prepared cell culture and sealed with Parafilm (M laboratory film, Sigma-Aldrich) and incubated as above. For the treatments at salinity 90 ppt, 18 replicates were inoculated to allow additional sampling in the latter part of the study, due to the predicted suppression of growth rate and hence elongation of the growth cycle, at high salinity. In addition, *C. closterium* was maintained in the dark (20°C, *n* = 3) at salinities 35, 70 and 90 ppt with 0 or 0.75% xanthan gum (w/v) and measured for population density only, to establish whether heterotrophic utilisation of EPS components occurred (Tuchman et al. 2006).

On the day of inoculation (day 0) and every one to three days after, the maximum quantum efficiency of PSII photochemistry (*F*_*v*_ */F*_*m*_) was measured in a sub-sample (three dishes) of each treatment. The cultures were dark-adapted for 30 min before measurement. The minimum (*F*_*o*_) and maximum (*F*_*m*_) fluorescence yields were measured with a Xenon-PAM (pulse amplitude modulated) fluorometer (HeinzWalz GmbH, Effeltrich, Germany) as described by Waring et al. (2006) with saturating light pulses (0.6 s) of ~ 4,000 μmol photons m^−2^ s^−1^ for measurement of *F*_*m*_. The ratio *F*_*v*_ */F*_*m*_ was calculated, where variable fluorescence *F*_*v*_ = *F*_*m*_ – *F*_*o*_ (Oxborough et al. 2000; Baker 2008).

On days 3, 8, 11 and 24 (also 29 and 37 for treatments at salinity 90 ppt) sub-samples of three dishes from each treatment were destructively sampled. Cells were counted using a haemacytometer. For the period of active growth, the specific growth rate, μ (day^−1^, Gotelli 1995), was determined for each treatment. The proportion of cells with compromised cell membranes (ie non-viable cells) was determined using the nucleic acid stain SYTOX-Green (Molecular Probes, S-34860, Life Technologies). SYTOX-Green was applied to a 1 ml sub-sample at a final concentration of 0.5 μM (Veldhuis et al. 2001) and incubated for 30 min in the dark, at culture temperature (conditions optimised prior to the study), before viewing cells *via* epifluorescence microscopy (Leitz, Ortholux II, Wetzlar, Germany). Total cells were counted under bright field, followed by SYTOX-Green stained cells under dark field. The proportion of live cells was calculated as population viability = (total no. of cells/no. of SYTOX-positive cells).

### Short-term response to salinity fluctuations

Cultures of *C. closterium* with starting population densities of 6.7 × 10^5^ cells ml^−1^, were grown in f/2 medium with 0 or 0.75% w/v xanthan gum (at standard seawater salinity of 35 ppt). Replicate (*n* = 5) cultures (1 ml) were grown in 5 ml wells (Sterilin, 25 well plates) sealed with Parafilm. After a growth period of 5 days, saline solution (1 ml, distilled water and NaCl) was applied to the top of the cultures. The applied solution was adjusted to produce final salinities within the cultures of 17.5, 35, 50, 70 and 90 ppt, giving a ‘salinity shock’.

The operating efficiency of PSII photochemistry (*F*_*q*_ *′/F*_*m*_ *′*) (Oxborough et al. 2000) was calculated before and after salt shock (at 15 s, 30 s, 1, 2, 4 and 6 min) to monitor changes in cell photosynthetic performance. Readings of *F′* (steady state fluorescence measured under actinic light) and *F*_*m*_ *′* (maximum fluorescence under actinic light) were taken at constant irradiance (5 ± 0.2 μmol photons m^−2^ s^−2^) 10 s before and 15 s, 30 s, 60 s, 2 min, 4 min and 6 min after treatment. The difference between *F*_*m*_ *′* and *F′,* (*F*_*q*_ *′* = *F*_*m*_ *′* – *F′*) and the ratio *F*_*q*_ *′/F*_*m*_ *′* were calculated. Maximum photosynthetic capacity (dark adapted *F*_*v*_ */F*_*m*_) was measured 24 h before, 1 h before and 24 h after the salt shock.

### Statistical analysis

Sample distributions were tested for normality and equal variance using the Anderson–Darling test and F-test respectively, conducted using Minitab software. All sample distributions in this study were positive for both normality (*p* > 0.05) and equal variance (*p* > 0.05); further parametric testing was applied using SPSS software. A two way analysis of variance (2-way ANOVA) test was used to detect differences between treatments with a *post hoc* Tukey test applied subsequently to determine differences between factors. A one way ANOVA was used when comparing between more than two levels of one variable (eg between the proportions of live cells at all salinities within one xanthan concentration). The *post hoc* Holm–Sidak method was then used to make all comparisons between all factors.

## Results

### Growth and viability at elevated salinities in a model EPS matrix

**Figure 1.**
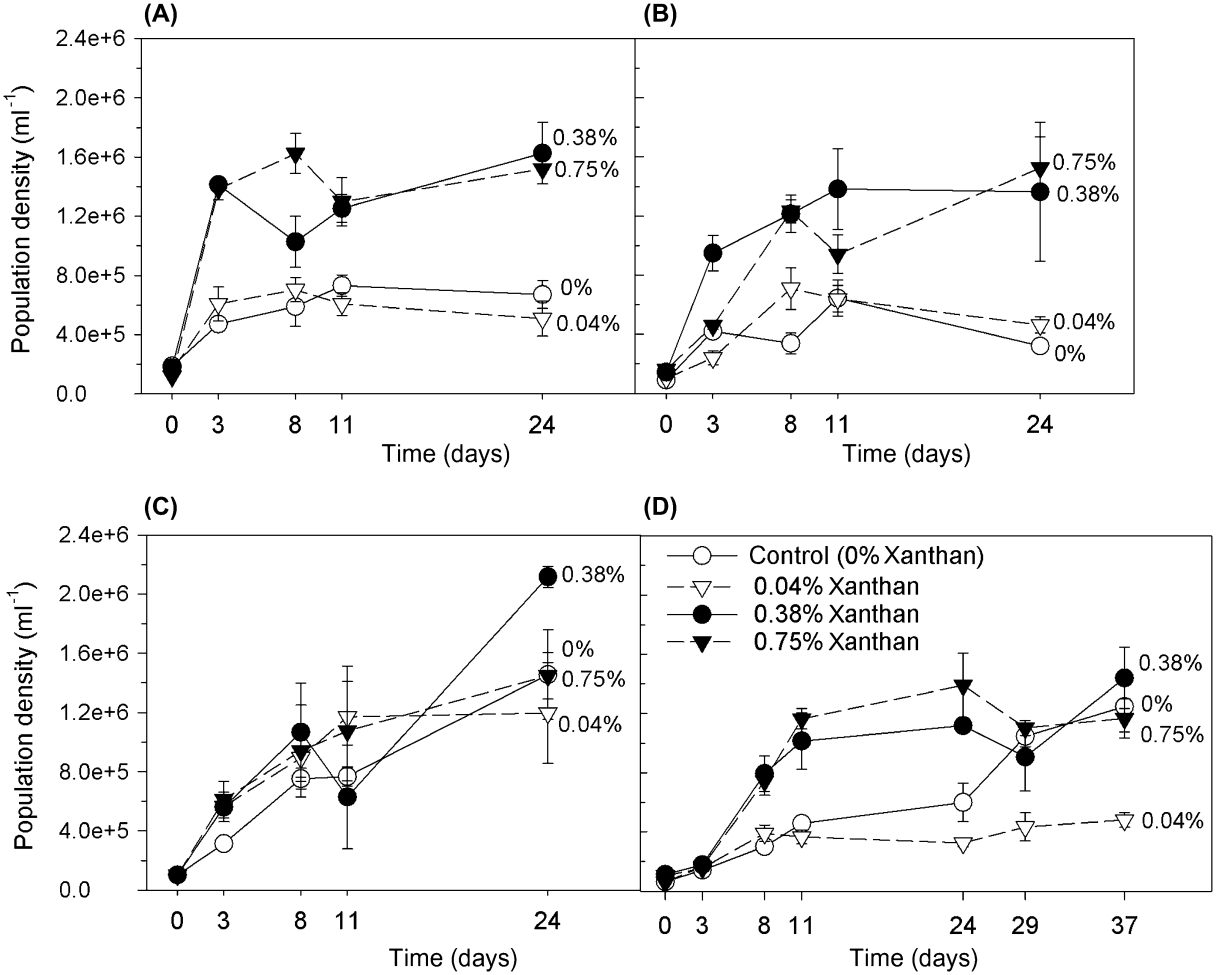
*C. closterium* culture population densities (ml^−1^) in salinities of (A) 35, (B) 50, (C) 70 and (D) 90 ppt (mean and SE, *n* = 3). The growth medium contained xanthan gum concentrations of 0, 0.04, 0.38 and 0.75%.

The maximum population density of *C. closterium*, attained during the growth cycle (day 0 to day 37) was significantly higher in cultures containing xanthan gum (0.38 and 0.75%), compared to those grown with low or no xanthan gum (0.04 and 0%) (Figure 1; 2-way ANOVA: F = 38.295, df = 3, *p* < 0.001, Holm–Sidak: *p* < 0.001). At salinities 35 and 50 ppt, growth in 0.38% and 0.75% xanthan gum more than doubled the maximum cell densities achieved compared to the controls and those in 0.04% xanthan gum (Figure 1). At salinities 35 and 50 ppt, population growth rates were significantly increased in cultures with 0.75% xanthan gum, compared to the controls and those in 0.04% xanthan gum (Table 1; ANOVA: F = 6.413, 61.055 with df = 3 and *p* = 0.05, Holm–Sidak: *p* < 0.05). Cultures incubated in dark conditions did not achieve any population growth (data not shown), hence the enhanced growth of cultures in high concentrations of xanthan gum was not due to heterotrophic utilisation of xanthan by the diatom cells.

**Figure 2.**
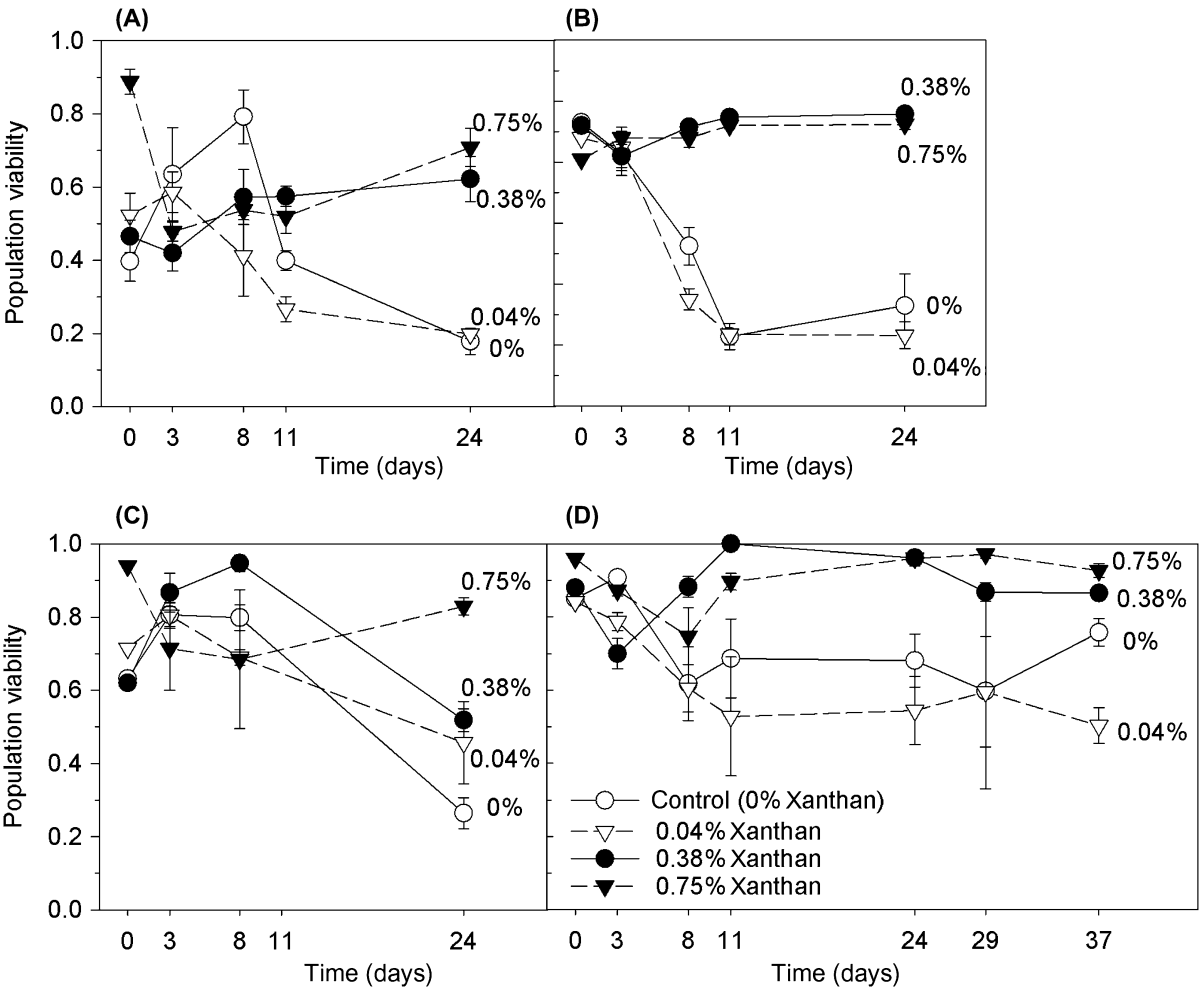
Proportion of live *C. closterium* cells (population viability) during growth in salinities of (A) 35, (B) 50, (C) 70 and (D) 90 ppt (mean and SE, *n* = 3). The growth medium contained xanthan gum at concentrations of 0, 0.04, 0.38 and 0.75%.

**Table 1.** Average specific growth rates, μ (d^−1^) for the period of active growth of *C. closterium* cultures (*n* = 3).

	Xanthan gum in medium w/v (%)	Active growth		
		Period (d)	μ	μ
			Mean (d^−1^)	SE
35	0	0–8	0.14	0.009
35	0.04	0–8	0.13	0.027
35	0.38	0–8	0.2	0.005
35	0.75	0–8	0.27	0.042
50	0	0–8	0.12	0.03
50	0.04	0–8	0.15	0.031
50	0.38	0–8	0.23	0.015
50	0.75	0–8	0.52	0.01
70	0	0–8	0.25	0.014
70	0.04	0–8	0.26	0.021
70	0.38	0–8	0.29	0.052
70	0.75	0–8	0.27	0.067
90	0	3–11	0.04	0.003
90	0.04	3–11	0.04	0.014
90	0.38	3–11	0.15	0.013
90	0.75	3–11	0.17	0.013

At the high salinities (70 and 90 ppt) the positive effect of xanthan gum (0.38 and 0.75%) on growth rate and maximum population density was not as conclusive as at the lower salinity (35 and 50 ppt) treatments. However, at salinity 90 ppt, growth rates were elevated in the 0.38% and 0.75% xanthan cultures, compared to those in 0.04% or no xanthan gum (Table 1; ANOVA: F = 273 with df = 3 and *p* = <0.001, Holm–Sidak: *p* < 0.001. Cells at this highest experimental salinity (90 ppt) without xanthan gum had an extended lag phase of 24 days, followed by a period of growth (Figure 1D) while those in 0.04% xanthan gum did not undergo an active growth period. Cultures at salinity 90 ppt in high levels of xanthan gum (0.38 and 0.75%) had short three-day lag periods followed by active growth. All cultures at salinity 70 ppt achieved relatively high maximum cell densities (Figure 1), compared with all other treatments, and all except those with 0.04% xanthan gum remained in active growth throughout the experimental period (Figure 1C) and growth rates were not significantly different between xanthan concentrations (Table 1; ANOVA: F = 0.149 with df = 3 and *p* = 0.927).

*C. closterium* grown in 0.38 and 0.75% xanthan gum concentrations maintained higher levels of population viability after 24 days (0.81 ± 0.06, mean ± SE, *n* = 8), compared to cultures grown in low or no xanthan gum (0.36 ± 0.06, Figure 2, ANOVA; F = 22.8 with df = 3 and *p* < 0.001). The largest effect on survival occurred at salinity 50 ppt where population viability was increased when the medium contained 0.75% xanthan gum, compared to no xanthan gum, equating to the survival of 60% more of the total population. At salinities 35 and 70 ppt, population viabilities were more than tripled in cultures containing 0.75% xanthan gum compared to no xanthan gum, from 0.18 to 0.71 and from 0.26 to 0.83, respectively (Figure 2). Differences were observed in the distributions of cells between xanthan treatments, regardless of salinity. *C. closterium* cells without xanthan gum grew as a lawn of cells on the bottom of the Petri dishes, spread out and attached. Cells in 0.38 and 0.75% xanthan gum grew in dispersed clumps suspended throughout the xanthan, suggesting that motility was curtailed, but not completely prevented, in this thickness of matrix.

### Effect of EPS on the photosynthetic efficiency of cells exposed to long-term salt stress

**Figure 3.**
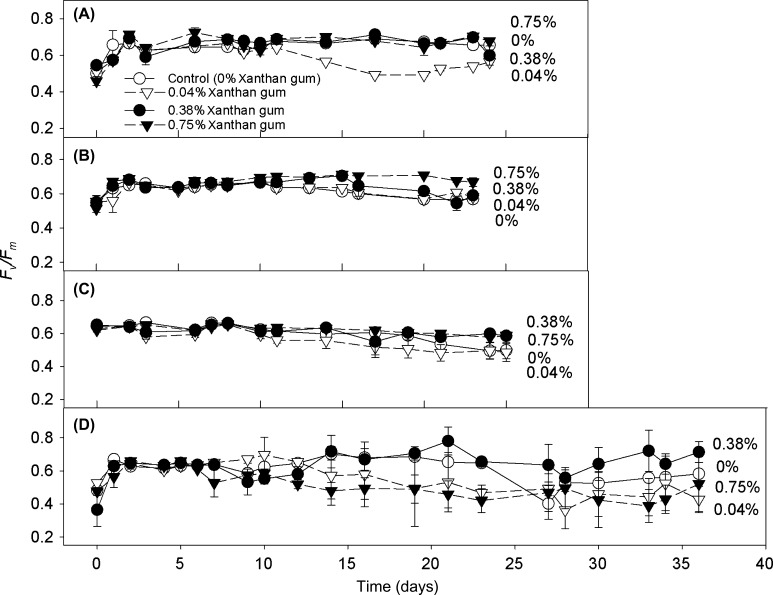
Dark-adapted *F*_*v*_ */F*_*m*_ of *C. closterium* cells grown at salinities (A) 35, (B) 50, (C) 70 and (D) 90 ppt and in medium containing xanthan gum concentrations of 0, 0.04, 0.38 and 0.75% (mean and SE, *n* = 3).

The degree and direction of the effect of xanthan gum on the maximum potential photosynthetic efficiency of PSII (*F*_*v*_ */F*_*m*_), of the *C. closterium* cultures was dependent on the concentration of the xanthan gum and on the salinity of the medium. The presence of xanthan gum had a protective effect on *F*_*v*_ */F*_*m*_ when cultures were grown in 0.75% xanthan gum, with *F*_*v*_ */F*_*m*_ significantly increased in cultures grown at salinities 50 and 70 ppt (Figure 3; ANOVA: F = 4.825, 3.594 with df = 3 and *p* = 0.05, Holm–Sidak*: p* < 0.009, 0.01). The presence of a low amount of xanthan gum (0.04%) had either a negative or negligible effect on *F*_*v*_ */F*_*m*_ (Figure 3). However at salinity 90 ppt, cultures grown in 0.38% xanthan gum had an increased *F*_*v*_ */F*_*m*_ (ANOVA: F = 7.807 with df = 3 and *p* < 0.001, Holm–Sidak: *p* < critical level).

### Protection of photosynthetic efficiency by xanthan gum during salinity fluctuations

**Figure 4.**
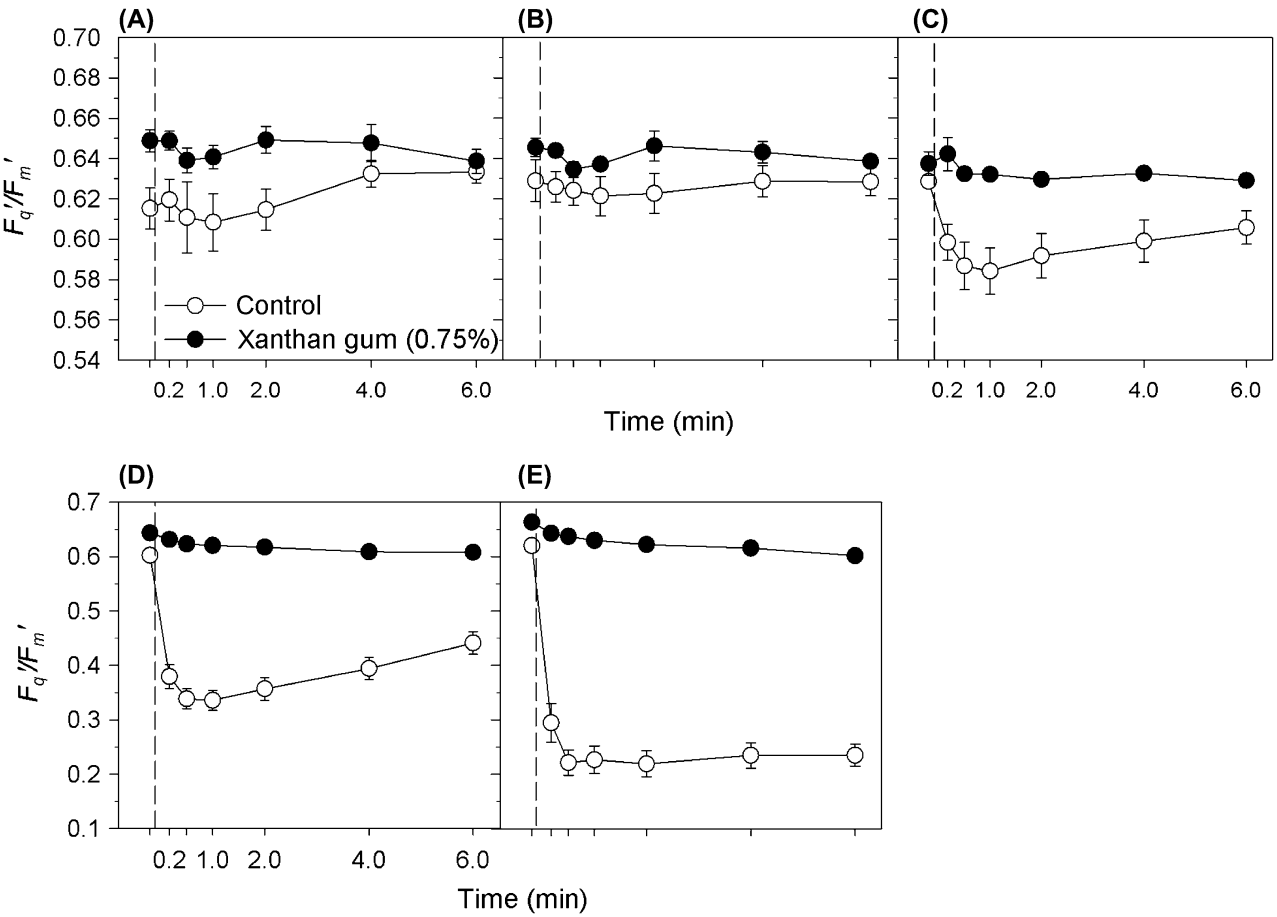
*F*_*q*_ *′/F*_*m*_ *′* response of *C. closterium* cultures grown at salinity 35 ppt in 0 and 0.75% xanthan gum when salt shocked to salinities (A) 17.5, (B) 35, (C) 50, (D) 70 and (E) 90 ppt. Mean and SE bars shown (*n* = 5). = time of shock (0 min).

**Figure 5.**
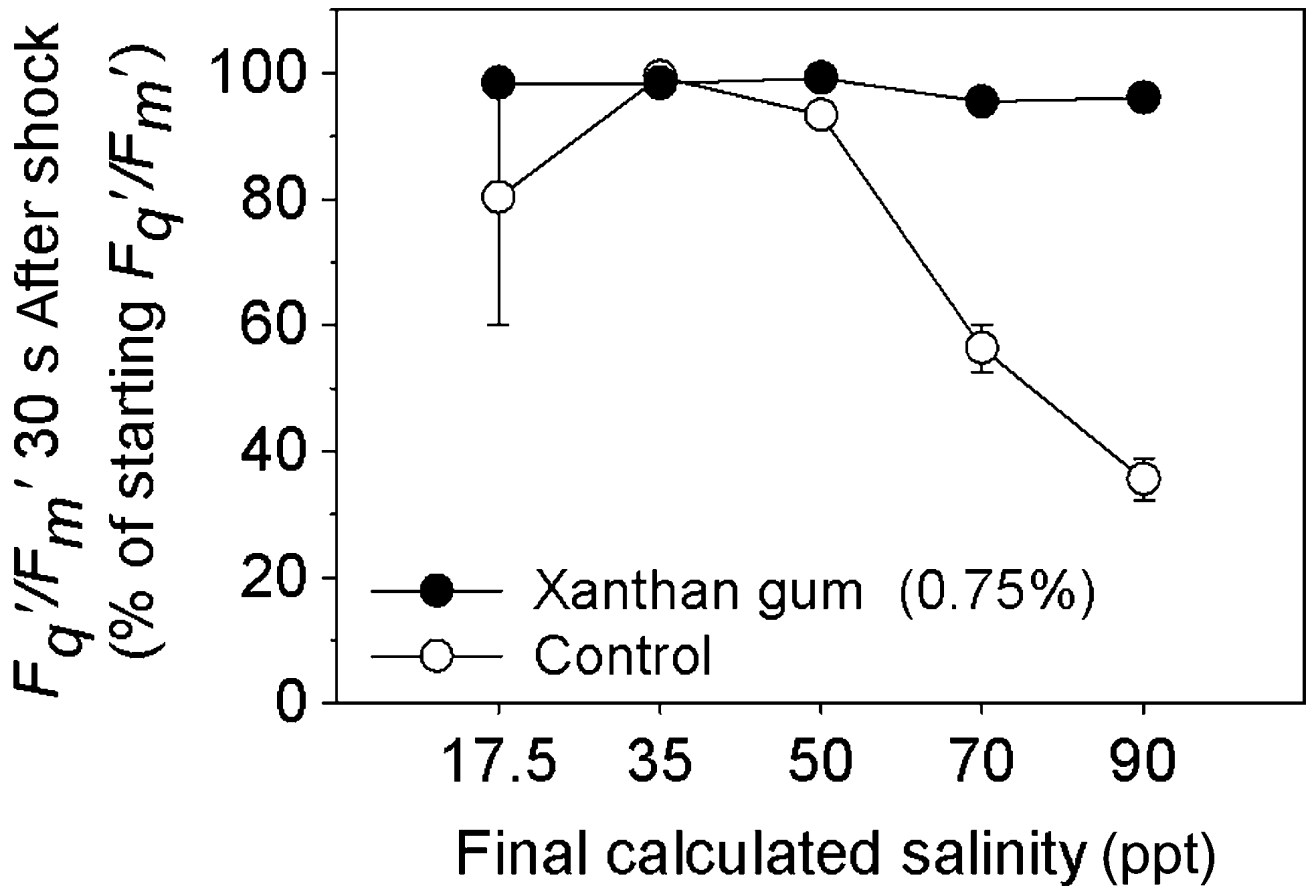
The initial percentage reduction in *F*_*q*_ *ʹ/F*_*m*_ *ʹ* in *C. closterium* cultures grown in 0% and 0.75% xanthan gum, 30 s after exposure to salinities of 17.5, 35, 50, 70 and 90 ppt (mean and SE, *n* = 5).

**Figure 6.**
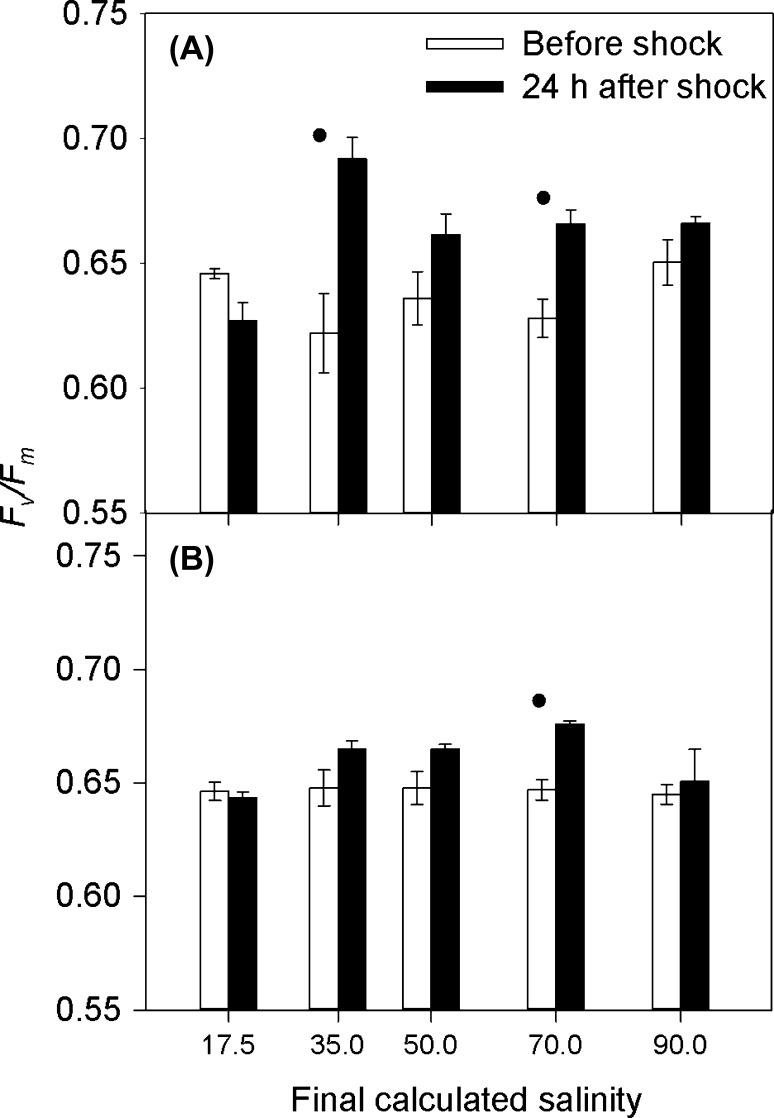
*F*_*v*_ */F*_*m*_ of *C. closterium* cultures grown in (A) 0% and (B) 0.75% xanthan gum. Means and SE bars are shown for *F*_*v*_ */F*_*m*_ before and 24 h after exposure to salt shock (of final salinities 17.5 to 90 ppt) (*n* = 5). 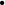 = a significant difference between the *F*_*v*_ */F*_*m*_ before and after the salinity shock (*p* < 0.05).

Cultures grown in 0 and 0.75% xanthan gum were subjected to an acute salinity shock by the application of saline solutions. The cultures grown in xanthan gum maintained high *F*_*q*_ *′/F*_*m*_ *′* (operating efficiency of PSII photochemistry) levels (Figure 4); between 96 and 100% of their original values (Figure 5) after the salinity shock was applied. In cultures grown in 0.75% xanthan gum a subtle decline in *F*_*q*_ *′/F*_*m*_ *′* occurred between 2 and 6 min after application. However, across all treatments, *F*_*q*_ *′/F*_*m*_ *′* did not fall below 97% of its original value (Figure 5). By 24 h after treatment, cultures treated with solutions of 50, 70 and 90 ppt salinity had increased *F*_*v*_ */F*_*m*_ (Figure 6A); the same response as cultures treated with the control solution at 35 ppt salinity (Figure 6B). This response was statistically significant at salinities 70 and 35 ppt only (*t*-test: *t* = –3.924, –3.881 with df = 6, 8 and *p* = 0.008, 0.005).

Cells grown in liquid medium (without xanthan gum), when exposed to a rapid change in salinity, suffered a steep decline in *F*_*q*_ *′/F*_*m*_ *′* (Figure 4) within 30 s of treatment. The level of decline in *F*_*q*_ *′/F*_*m*_ *′* was dependant on the magnitude of the salinity change (Figure 5), with the largest decrease observed after application of solution at salinity 90 ppt (65%). Recovery of *F*_*q*_ *′/F*_*m*_ *′* was observed (Figure 4), with recovery time increasing with the increasing salinity of the treatment (Figure 4). By 24 h after treatment, the *F*_*v*_ */F*_*m*_ of all cultures had returned to their original level (as before treatment), with the exception of salinity 70 ppt where *F*_*v*_ */F*_*m*_ increased (Figure 6B). Therefore the maintenance of *F*_*v*_ */F*_*m*_ was not dependent on growth in xanthan gum.

## Discussion

Natural biofilms consist of a heterogeneous range of microbial taxa, chemical constituents and physical structures (Dobretsov et al. 2013; Mieszkin et al. 2013). The complexity present in natural biofilms provides challenges in determining the exact nature of cell–cell and cell–environment interactions, though broad scale patterns can be determined. Experimental studies using simplified systems have been used successfully to demonstrate species-specific positive and negative interactions between bacteria and diatoms, EPS production and concentrations, cell settlement and environmental conditions such as flow, carbon cycling and salinity conditions (Bruckner et al. 2011; Krembs et al. 2011; Aslam et al. 2012; Taylor et al. 2013; Zargiel & Swain 2014). The reductionist approach (a model system) was used in the present study to assess the role of EPS in protecting a biofilm-forming diatom during elevated and fluctuating salinities. EPS is a key characteristic of diatom-dominated biofilms (Underwood & Paterson 2003), and the results presented here demonstrate that the presence of an EPS matrix leads to a higher prevalence of viable cells (cells which have the capacity to divide in the future) in biofilms undergoing elevated and fluctuating salinities, such as would be experienced on ships’ hulls, in sea-ice and intertidal benthic environments. A natural biofilm contains an EPS matrix consisting of polymers from multiple sources (algal, bacterial, fungal, animal) and can be subject to various levels of both production and degradation by heterotrophic organisms (Dobretsov et al. 2013; Taylor et al. 2013). Such complexity is not possible to replicate under control conditions, but xanthan gum is widely used and accepted as an analogue for EPS in many studies (Passow & Alldredge 1995; Hart et al. 2001; Krembs et al. 2011; Aslam et al. 2012). Therefore EPS (represented by xanthan gum) appears to be an important adaptation which promotes population persistence for diatom biofilms, as has been previously demonstrated in bacterial biofilms.

Very few studies on diatom biofilm EPS production and composition also include measurements of the potential growth benefits of being in an EPS matrix. The experimental design used here aimed at removing as many other possible variables known to effect diatom physiology and EPS production, such as light–dark cycles and varying irradiance levels. This enabled the focus to be on the impacts of the EPS matrix on cell growth and viability. The growth rates and photosynthetic parameters of *C. closterium* reported here are very similar to those reported from a range of other studies (Smith & Underwood 2000; Staats et al. 2000; Underwood et al. 2004; Waring et al. 2006). This provides confidence in the design of the presented model system, and shows that there are benefits to cells of growing in an EPS matrix when challenged with salinity stress. However, the elevated growth rates observed with the addition of EPS at standard salinity in this study are in contrast to results from a sea-ice diatom, *Fragilariopsis cylindrus*, where growth rate was unaffected by addition of 0.1 and 0.5% xanthan gum at standard (34 ppt) and elevated (52 ppt) salinities (Aslam et al. 2012). However, for *C. closterium* at standard and elevated salinities, the growth rate was increased by the addition of xanthan gum at 0.38 and 0.75%, respectively. The two studies are not directly comparable, as in the Aslam et al. (2012) study *F. cylindrus* cultures at salinities 34 and 52 ppt at 0°C had a significant pre-experimental acclimation period. Growth rates of *F. cylindrus* at salinities 34 and 52 ppt were 0.25 d^−1^ ± 0.03 and 0.13 d^−1^ ± 0.133 respectively (Aslam et al. 2012), which are comparable to growth rates of *C. closterium* at standard (35 ppt) salinity containing 0.38% xanthan gum and elevated (50 ppt) salinities without xanthan gum (0.2 d^−1^ ± 0.005 and 0.12 d^−1^ ± 0.03 respectively). Aslam et al. (2012) found that addition of xanthan gum inhibited freezing of the medium during stepwise decreases in temperature from 0°C to –12°C. *F. cylindrus* at standard salinity without xanthan gum continued to grow at –4°C. However, growth was suppressed in cultures with 0.1% xanthan gum. *F. cylindrus* with 0.5% xanthan gum continued to grow at –4°C and freezing was avoided until the temperature was reduced to –12°C (Aslam et al. 2012). The differences in the level of ‘growth enhancement’ due to the presence of xanthan gum between these studies may therefore reflect an interplay between the presence of an EPS matrix, the salinity of the medium and ice formation in the low temperatures tested by Aslam et al. (2012). There may also be species or habitat specific responses to living in xanthan gum. In general, sea-ice diatoms are more acclimated to changes in salinity due to seasonal changes with temperature (Ewert & Deming 2013). Aslam et al. (2012) found that *F. cylindrus* produced proportionally higher amounts of complex EPS; hence such species may be pre-adapted to salinity changes.

Other studies on algal EPS have concentrated on factors affecting production (Wolfstein & Stal 2002) and analysis of structure (Mishra & Jha 2009). Hence the findings of elevated population viability and maximum population densities with the addition of xanthan, at standard salinity, provides the first evidence for benthic diatoms that being embedded in an EPS matrix is beneficial to growth regardless of salinity. The mechanistic basis of this benefit may be similar to the EPS capsule of bacteria, where nutrients and exoenzymes are concentrated close to the cell membrane (Tonn & Gander 1979), thus facilitating faster growth. However, at standard salinity the presence of xanthan gum at a low concentration of 0.04% had a detrimental effect on *F*_*v*_ */F*_*m*_, and at high salinity (90 ppt) it also had a detrimental effect on growth and viability. There may be a particular level of matrix density that is required for the physical benefits of being in a biofilm to manifest themselves. Below this threshold, at very low EPS concentrations, potential negative effects include reduction in nutrient diffusion to the cells, and the inhibition of movement. These concepts were not addressed in this study and require further investigation.

The beneficial effects of EPS were greater at the higher salinities tested in this study. However, the type and level of physiological benefit provided by xanthan (at 0.75 and 0.38%) varied with its salinity, with population density, growth rate and viability most enhanced at salinities 35 and 50 ppt, when *F*_*v*_/*F*_*m*_ was also maintained. At salinity 70 ppt, *F*_*v*_/*F*_*m*_ was enhanced by xanthan gum (at 0.75 and 0.38%). However, population density and viability data were inconclusive. Cultures at salinity 90 ppt had a similar growth curve, after a three-day lag period, as control cells (at salinity 35 ppt) providing xanthan was present at 0.75 or 0.38% but viability was increased with the presence of xanthan gum. Salinity of 75 ppt is a known upper limit for growth in many benthic diatom species (Clavero et al. 2000) and natural benthic microalgal diversity is known to decrease at salinities above 75 ppt (Herbst & Blinn 1998). Hence resource allocation was similar at salinities 35 and 50 ppt but a change in allocation occurred at salinities 70 and 90 ppt.

Ambient salinity is known to affect the quantity and composition of EPS in diatoms (Allan et al. 1972; Abdullahi et al. 2006; Apoya-Horton et al. 2006). When acclimated to elevated salinity (52 ppt) the sea-ice diatom *F. cylindrus* produced around three times more EPS than at standard salinity (34 ppt) (Aslam et al. 2012). *C. closterium* cells may have changed the composition of their EPS. This is a known response to hyper-salinity and an adaptation to resist freezing in brine channels during sea-ice formation when growth is limited (Krembs & Deming 2008), for example in *Phaeodactylum tricornutum* (Abdullahi et al. 2006) and *Nitzschia frustulum* (Allan et al. 1972). It was not possible to test this in the experiments presented due to the masking nature of the xanthan in the medium. EPS used for motility is also altered during hypo- and hyper-saline conditions in *C. closterium* (Apoya-Horton et al. 2006), which is known to affect diatom migration (Sauer et al. 2002).

Living in xanthan gum allowed the operating efficiency of PSII photochemistry (*F*_*q*_ *′/F*_*m*_ *′*) of *C. closterium* cells to be maintained during salinity shock; hence it is assumed that the photosynthetic apparatus was protected, most likely through forming a physical barrier around cells. Living in an EPS matrix when the external environment is undergoing salinity changes will slow the permeation of salt and osmotic stress to embedded cells. The mechanical action of forming a physical diffusion barrier is a known function of EPS in sea ice, where brine channels are ‘plugged’ by the EPS (Krembs & Deming 2008). If that change in salinity is transient then cells within the gel will not have used energy acclimating to a changing external salinity environment. However, if the altered salinity is permanent the gel would mediate the penetration of ions, so reducing osmotic stress to the cells and thereby the amount of energy used in protective mechanisms. In addition, an EPS matrix may collect and concentrate compatible solutes close to the cells which would help to retain water activity, stabilise macromolecules and aid osmoregulation (Yancey et al. 1982), allowing the maintenance of cellular function during salinity shocks. Under hypo-saline conditions, diatoms expel compatible solutes into the environment, which are retained by the EPS matrix and can be re-imported during hyper-saline conditions, thus avoiding the metabolic cost of synthesis. For example in hypo-saline conditions, *C. closterium* rapidly excretes dimethylsulfonioproprionate (DMSP) (Nilsson & Sundbäck 1996; Van Bergeijk et al. 2003) and amino acids (Admiraal et al. 1984) into the surrounding EPS. A possible mechanism for the release and import of compatible solutes is a change in the selective permeability of the cytoplasmic membrane caused by an altered environmental salinity, with the level of permeability depending on the magnitude of the salinity change (Schobert 1980). Regulation of compatible solutes by export and import is thought to be a mechanism of short term osmo-acclimation for diatoms living in the highly fluctuating salinities of intertidal sediments (Van Bergeijk et al. 2003).

The results presented also demonstrate that the development of a diatom biofilm under normal growth conditions can help cells to maintain photosynthetic function when subjected to salinity shocks such as those found in intertidal sediments during sea ice development, permanent structures in tidal estuaries, and in maritime shipping. It is also highly likely that other organisms (eg bacteria, fungi and macroalgal sporelings; Dobretsov et al. 2013; Mieszkin et al. 2013) living within photosynthethic biofilms benefit from the EPS matrix predominantly produced by diatoms. Of particular interest is the idea that cell lysis and local decomposition of the matrix creates pores allowing better nutrient transport to the cells (Webb et al. 2003). Aggregates of dead cells were observed in medium containing xanthan gum, indicating mechanistic control of cell death. A relationship between cell aggregation and population viability has also been reported in the diatom *Thalassiosira weissflogii*; aggregation prolonged high population viabilities in dark, nutrient-limited conditions (Garvey et al. 2007). Quorum sensing, a cell–cell communication mechanism used by Gram-negative bacteria is known to help regulate certain components of EPS (De Kievit 2009; Borlee et al. 2010) and an adaptive role for diatom cell death could potentially be investigated in the *C. closterium* model EPS system developed here.

In conclusion, this study has demonstrated for the first time that the presence of EPS can increase the viability, maximum population density and growth rate of diatom populations in biofilms in standard and elevated salinities. *C. closterium* is a cosmopolitan diatom species, widely distributed in biofilms on natural silts, sand, rocks, sea ice, biotic and artificial substrata and has a similar photophysiology and EPS physiology to many of the other widespread biofilm-forming diatom genera, eg *Achnanthes*, *Nitzschia* and *Navicula* (Underwood & Paterson 2003; Chiovitti et al. 2004; Bellinger et al. 2005; Apoya-Horton et al. 2006). These taxonomic similarities, and the similarity in the emergent properties of mature biofilms (resilience, intense microbial coupling, and productivity), despite both structural and taxonomic diversity (Decho 1990; Dobretsov et al. 2013; Mieszkin et al. 2013) suggest that the beneficial properties of being embedded in an EPS matrix for cell growth, viability and photosynthesis described here may be applicable to marine photosynthetic biofilms in general.
